# The Effect of Organic and Conventional Cultivation Systems on the Profile of Volatile Organic Compounds in Winter Wheat Grain, Including Susceptibility to Fusarium Head Blight

**DOI:** 10.3390/metabo13101045

**Published:** 2023-09-30

**Authors:** Maciej Buśko, Anna Gracka, Henryk Jeleń, Kinga Stuper Szablewska, Anna Przybylska-Balcerek, Lidia Szwajkowska-Michałek, Tomasz Góral

**Affiliations:** 1 Department of Chemistry, Poznań University of Life Sciences, 60-625 Poznań, Poland; maciej.busko@up.poznan.pl (M.B.); kinga.stuper@up.poznan.pl (K.S.S.); lidia.szwajkowska@up.poznan.pl (L.S.-M.); 2 Food Volatilomics and Sensomics Group, Faculty of Food Science and Nutrition, Poznań University of Life Sciences, 60-624 Poznań, Poland; anna.gracka@up.poznan.pl (A.G.); henryk.jelen@up.poznan.pl (H.J.); 3 Plant Breeding and Acclimatization Institute-National Research Institute, 05-870 Radzików, Poland; t.goral@ihar.edu.pl

**Keywords:** volatiles, organic farming, conventional farming, winter wheat, resistance

## Abstract

The grain of 30 winter wheat cultivars differing in terms of their resistance to FHB (Fusarium head blight) was tested. The cultivars were grown in four variants of field trials established in a split-plot design: control without fungicides, chemical control of FHB with fungicides after *Fusarium* inoculation, *Fusarium* head inoculation, and organic cultivation. The profile of volatile compounds in grain samples was determined by mean headspace–solid phase microextraction and analyzed by gas chromatography time-of-flight mass spectroscopy. The identified volatile profile comprised 146 compounds belonging to 14 chemical groups. The lowest abundance of volatile organic compounds (VOCs) was found for the organic cultivation variant. The performed discriminant analysis facilitated the complete separation of grain for individual experimental variants based on the number of VOCs decreasing from 116 through 62, 37 down to 14. The grain from organic farming was characterized by a significantly different VOCs profile than the grain from the other variants of the experiment. The compounds 1-methylcycloheptanol, 2-heptanone, 2(3H)-furanone, and 5-hexyldihydro-2(3H)-furanone showed statistically significant differences between all four experimental variants.

## 1. Introduction

Cereals play a dominant role in world plant production. Among them, wheat is one of the most important—global wheat production has reached 802 million tons in 2023 (OECD/FAO, 2023). Wheat grain is mainly used for human consumption, with animal feed and industrial uses ranking next [1]. Factors affecting the quality and safety of wheat grain include, e.g., tillage system and applied cultivation measures such as the use of fungicides [2]. Recently, both the authors of this study and other researchers [3] have been observing the development of alternative farming systems. Next to conventional and integrated farming, a significant role is played by organic farming [3]. The reduction of chemical treatments in cultivation is in line with consumer expectations. In terms of the safety of raw materials and cereal products, it is essential to undertake actions to prevent the development of pathogens, particularly those producing metabolites toxic to humans and animals. Among wheat pathogens found in the temperate climate zone, fungi from the genus *Fusarium* play a significant role in causing head blight. This disease causes a deterioration of grain quality and reduced yields. Grain infested by fungi from the genus *Fusarium* is contaminated with their secondary metabolites, i.e., mycotoxins from the group of trichothecenes, which greatly affect the health safety of cereal products [4].

In conventional agriculture, the use of fungicides provides significant protection against mass-scale fungal infections and the resulting mycotoxin contamination of grain [5]. In contrast, organic agriculture, in which no chemical pesticides are applied, uses alternative antimicrobial methods and cultivation measures [6]. For this reason, organic farming has been gaining an advantage over conventional agriculture since natural plant resistance mechanisms are induced [7]. Additionally, the absence of chemical pesticides does not disturb the natural homeostasis of kernel surface microflora (phyllosphere), thus naturally limiting the development of pathogens thanks to the natural competition between microorganisms [8]. All the above-mentioned factors affect the quality attributes of cereal grain. Although grain produced in organic farming systems is frequently smaller and its yield is lower compared to conventional agriculture, the quality and health value of organic grain is significantly superior, thus compensating for the above-mentioned drawbacks [9,10].

One of the quality attributes of grain relates to its aroma [11]. The profile of volatile compounds may also indicate microbiological changes taking place on grain surface (or more broadly speaking in the phyllosphere) [3]. It also strongly suggests the presence of pathogenic toxin-forming fungal strains [12].

The volatile organic compounds (VOCs) are low molecular weight compounds produced in both primary and secondary metabolism. Additionally, their analysis in cereal grain samples shows that these compounds originate not only from plant and microbial pathways but also from the activity of plant enzymes on microbial (mainly fungal) substrates and in turn microbial enzymatic activity on plant substrates. Generally, it can be stated that grain VOCs are products of plant metabolism, microbial metabolism, and the effect of their interactions. As reported by Knudsen et al. [13], over 1700 VOCs originating from 90 plant families have been identified up to now. Although plant VOCs are products of primary and secondary metabolism, all of them can be derived from only one of the following compounds: erythrose-4-phosphate, farnesyl pyrophosphate, pyruvate, and acetyl-CoA [14]. In the shikimate pathway, erythrose-4-phosphate along with phosphoenolpyruvate are converted through chorismate to aromatic compounds, including aromatic amino acids. In further steps, the biosynthetic pathways lead to such biochemical groups as terpenoids, phenylpropanoids/benzenoids, derivatives of fatty acids and amino acids, etc. Microbial VOCs can also be produced during primary and secondary metabolism [15]. Microbial VOCs include such compounds as alcohols, ketones, aldehydes, terpenes, esters, lactones, and hydrocarbons, as well as sulfur and nitrogen compounds [16]. The sources of the microbial VOCs, similar to plant VOCs, may be amino acids, fatty acids, or isopentenyl pyrophosphate [15]. 

The available literature on the subject contains no reports on the variability of the VOCs profile, depending on the adopted cultivation system, particularly concerning organic farming. Given the above, it was decided to verify whether the cultivation system may significantly affect the profile of produced VOCs.

This study aimed to compare the profiles of VOCs and to identify compounds differentiating cultivation systems, focusing on metabolites of pathogenic fungi from the genus *Fusarium* in grain produced in conventional and organic farming. The experiment was designed for the grain of the 30 wheat cultivars most grown in Europe.

## 2. Material and Methods

### 2.1. Field Experiments

Thirty cultivars of winter wheat (*Triticum aestivum* L.) were evaluated. The cultivars are listed in the Polish National List of the Research Center for Cultivar Testing (COBORU) and were added to the list between 1998 (‘Mewa’) and 2009 (‘Belenus’). The cultivars were described in detail in the paper by Góral et al. [17]. The cultivars differed in their morphological characters, resistance to diseases, and pedigree. Cultivars were grouped into four classes of Fusarium head blight (FHB) resistance: susceptible (S), medium susceptible (MS), medium resistant (MR), and resistant (R) (Table 1).

Field experiments were established in 2020 in the experimental fields of the Plant Breeding and Acclimatization Institute (IHAR-PIB) in Radzików, Central Poland. The field trial was established using a split-plot design, where 1 m^2^ plots were designated for planting different wheat cultivars. The experiment included four treatments (blocks): a control group without fungicide treatment and inoculation (C), chemical control using fungicides to manage FHB (F) which was induced by artificial inoculation, head inoculations (I) without chemical control, and an organic treatment (O) without inoculation. Every block consisted of three replicates. 

For treatments F and I, the same set of *Fusarium culmorum* isolates were applied. In the organic treatment, wheat was cultivated following organic farming practices, excluding chemical disease control and synthetic fertilizers. 

Three blocks (C, F, I) were sown in the conventional field (GPS coordinates: 52.21131, 20.63133). The soil was rich sandy clay of class 3 (according to soil quality classification in Poland). Pre-crop was oilseed rape. Artificial fertilizers were applied according to standard agricultural practices. In the autumn, 3 dt ha^−1^ of ‘Polifoska 6’ fertilizer was applied (N—18 kg ha^−1^, P—45 kg ha^−1^, K—72 kg ha^−1^) (Grupa Azoty, Zakłady Chemiczne Police S.A., Police, Poland). In the spring, after the start of vegetation, ammonium nitrate fertilizer (Grupa Azoty, Zakłady Azotowe Puławy S.A., Puławy, Poland) was applied in an amount providing 68 kg N ha^−1^. Weeds and pests were controlled with herbicides and insecticides. Immediately after sowing, weeds were controlled with the herbicide ‘Maraton 375SC’ (BASF SE, Ludwigshafen, Germany) (isoproturon + pendimethalin) in a dose of 4 L ha^−1^. In spring, weeds were controlled using the herbicide ‘Attribut 70GS’ (Bayer CropScience AG, Monheim, Germany) (propoxycarbazone-sodium) in a dose of 60 mg ha^−1^. Cereal leaf beetle and aphids were controlled with ‘Cyperkill Max 500 EC’ (Arysta LifeScience, Ougre, Belgium) (cypermethrin) in a dose of 50 mL ha^−1^. No fungicides were applied.

The fourth block (O) was sown in the experimental organic field of IHAR-PIB (GPS coordinates: 52.21706, 20.63827). The soil was rich sandy clay of class 2 (according to soil quality classification in Poland). The field has a valid organic farming certificate and has been cultivated using organic methods since 2013. Pre-crop was lacy phacelia. Weeds were controlled mechanically. Fertilizers allowed in organic farming were applied in the autumn. They were: ‘Fertil 12.5’ (C-N 40-12.5) (NaturalCrop, Warszawa, Poland) and ‘Nawóz Ekologiczny 0-8-18’ (N-P-K 0-8-18; Mg-S 8-12) (Luvena, Luboń, Poland). Both fertilizers were applied at 500 kg ha^−1^.

Sowing occurred from the last week of September to the first week of October. Wheat was sown using a plot seeder PlotseedTC (Wintersteiger, Ried/I, Austria) with 6 rows per plot. Conventional tillage was applied in both fields at the end of August. The distance between the two experimental fields was about 500 m. 

To manage FHB, two fungicides were applied: ‘Prosaro 250 EC’ (Bayer CropScience), consisting of tebuconazole (125 g/L) and prothioconazole (125 g/L), applied during the heading stage (BBCH 55-59) [18]; and ‘Topsin M’ (Sumi Agro, London, UK), containing thiophanate methyl (500 g/L), applied after flowering at stages BBCH 69-71.

To produce inoculum, three *Fusarium culmorum* isolates that produced deoxynivalenol (DON)-KF846, ZFR112, and nivalenol (NIV)-KF350, were applied. Details of isolates origin and identification were described in papers of Góral et al. [19,20]. Isolates were stored in 10% (*v*/*v*) glycerol at −70 °C in an isolate collection of IHAR-PIB, Radzików. Before use, isolates were subcultured onto potato dextrose agar (PDA) medium (Carl Roth GmbH, Karlsruhe, Germany) and incubated at 20 °C. Inoculum of *F. culmorum* isolates was produced on autoclaved wheat grains. A measure of 30 g of wheat grain was placed in a 300 mL Erlenmeyer flask and 30 mL of distilled water was added. Flasks were autoclaved twice for 30 min at 120 °C on two consecutive days. Grain was inoculated with 6–7 PDA agar discs (⌀ 1 cm) with *F. culmorum* mycelium. Flasks with inoculated grain were incubated for 7 days in darkness at 18 °C, followed by exposure to near UV light (360 nm) under a 16-h photoperiod for 3 weeks at 15 °C. UV light was applied to stimulate the sporulation of *F. culmorum*. The source of UV light was Phillips TL-D 36W BLB 1SL/25 blacklight lamps. Flasks were shaken thoroughly daily to loosen kernels colonized with mycelium. The mycelium-colonized grains were then air-dried and stored at 4 °C in a refrigerator until use.

For inoculation, the *Fusarium* mycelium-infused grains were suspended in tap water for 2 h and filtered to create a conidial suspension separately for each isolate. Concentrations of the suspensions from all the isolates were adjusted to 5 × 10^5^ spores/mL. Measurements of spore concentration were made using a Thoma hematology chamber (VWR International Sp. z o.o., Gdańsk, Poland). The equal volumes of spore suspensions of three isolates were mixed. 

During anthesis (BBCH 65), wheat heads were sprayed with spore suspension at a rate of 100 mL/m^2^. Inoculation was repeated 3 days later. The effectiveness of the inoculations was evaluated by assessing FHB severity. After maturation, 50 randomly chosen heads from each plot were manually harvested and threshed using a laboratory thresher with low wind speed to prevent loss of lightweight infected kernels. Visual inspection was applied to assess kernel damage caused by *Fusarium*.

Weather conditions in 2020 were favorable for FHB development (Table 2). Precipitation was high during the heading (May), flowering of wheat (I decade of June), and after flowering (II decade of June). In III decade of June, the sum of precipitation was low; however, rainfall occurred with high frequency.

### 2.2. Analysis of VOCs

Extraction and analysis of volatiles from grain samples was performed as in Gracka et al. [21]. Volatiles from the grain samples were extracted by solid phase microextraction method (SPME) using carboxene/divinylbenzene/polydimethylsiloxane (CAR/DVB/PDMS) 2 cm fiber (Supelco, Bellefonte, PA, USA). Then, 6 g of each sample was placed in a 10 mL glass vial and kept in a heating block at 50 °C for 5 min to equilibrate. Subsequently, the volatiles were extracted from the headspace at 50 °C for 30 min. After the extraction, the SPME fiber was desorbed for 5 min into the GC injector at 250 °C. The VOCs were analyzed by comprehensive two-dimensional gas chromatography-time of flight mass spectrometry (GC × GC-ToFMS) on Agilent 6890N gas chromatograph (Agilent Technologies, Palo Alto, CA, USA) equipped with ZOEX cryogenic (N2) modulator coupled to PEGASUS 4 time-of-flight mass spectrometer (LECO, St. Joseph, MI, USA). The samples were injected by Agilent Technologies GC Sampler 80 autosampler with SPME capabilities. GC × GC extends the chromatographic separation by pairing two columns with complementary stationary phases. Analytes that coelute with one type of stationary phase do not necessarily coelute on a different type of stationary phase. The compounds were resolved by a nonpolar-polar column system: DB-5 (30 m × 250 µm × 0.1 µm) as a first-dimension column and Supelcowax-10 (0.75 m × 100 µm × 0.1 µm) as a second dimension column. All injections were performed in a splitless mode. The carrier gas was helium at a constant flow rate of 0.8 mL/min. The operating conditions of the first column were the following: initial oven temperature 45 °C (1 min), 6 °C/min to 175 °C (0 min), and 25 °C/min to 245 °C (5 min). The second column was programmed from 60 °C (1 min), 6 °C/min to 190 °C (0 min), and 25 °C/min to 260 °C (5 min). The injector temperature was 250 °C, whereas the GC/MS transfer line was set at 270 °C. The modulation time was 4 s. The time-of-flight mass spectrometer was operated at a mass range of *m*/*z* 38–388 and detector voltage 1700 V at 150 spectra/s. The data were collected and processed using LECO^®^ChromaTOF^®^v.4.40. 

### 2.3. Statistical Analysis

Statistical analysis was performed using Microsoft^®^ Excel 2010/XLSTAT©-Pro (Version 2012.6.09, Addinsoft, Inc., Brooklyn, NY, USA). To compare contents of VOCs in samples, Tukey’s multiple comparison procedure was used, with identical letters in rows or columns denoting a lack of differences at the significance level *p* = 0.05. Step linear discriminatory analysis (SLDA) was used to separate groups of analyzed populations.

## 3. Results

**Figure 1:**
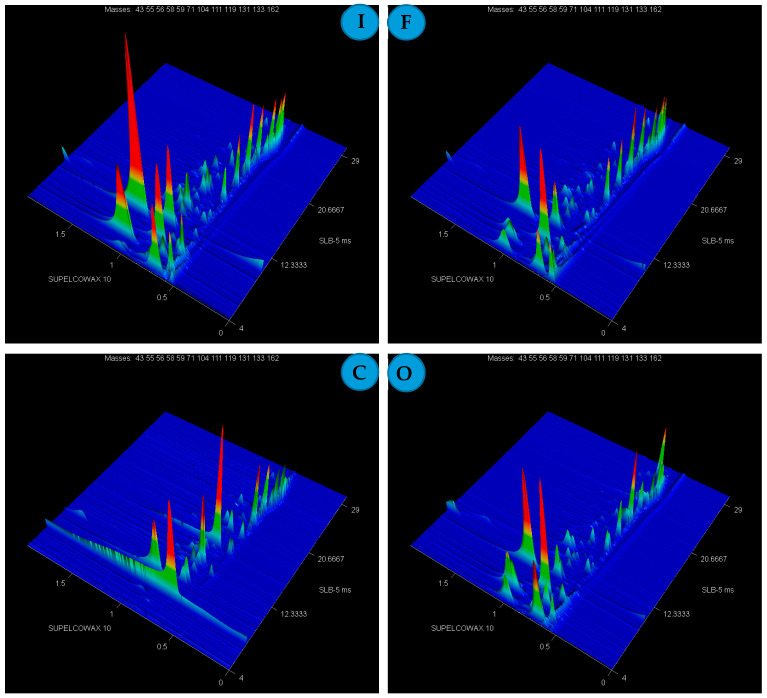
GC × GC-ToFMS chromatogram of volatile compounds obtained from wheat grain (cultivar Kampana) from inoculated (I), fungicide (F), control (C), and organic (O) variant of the experiment. The same peak intensity scale is used for all chromatograms.

Analyses of VOCs were conducted in the grain of 30 wheat cultivars grown in 4 experimental variants presented in the Materials and Methods section, i.e., the control (C), organic farming (O), inoculated (I), and inoculated and protected with a fungicide (F). Analyses of VOCs from the headspace in SPME GC × GC ToFMS in all samples identified 146 VOCs. Example chromatograms for all experimental variants are presented in Figure 1 for cv. Kampana. 

**Figure 2:**
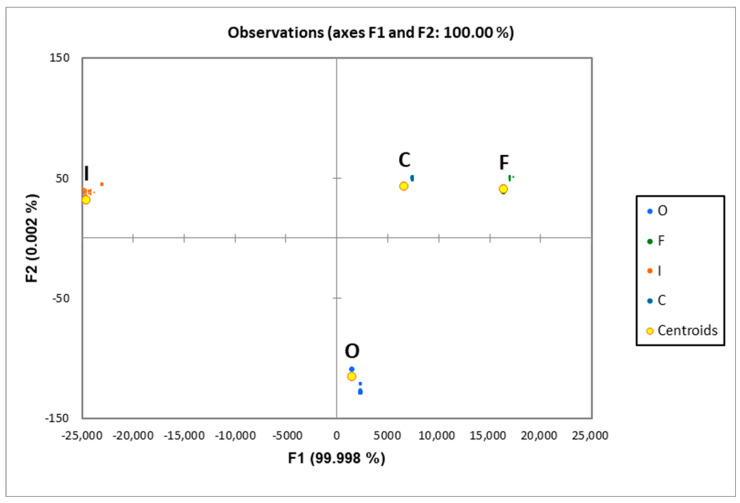
Results of discrimination analysis based on a model including 116 out of 146 identified VOCs. (Centroids of particular groups—average of observations of 30 analyzed varieties according to Table 1; O—organic, F—fungicide, I—inoculated, C—control).

It was found that individual cultivars did not differ within an experimental variant (O, F, I, C). In contrast, significant differences were observed between experimental variants. Overall, the highest abundance was recorded in the inoculated samples, it was lower for the fungicide treatment and control, while it was lowest in the organic farming samples. The identified compounds belonged to 14 different chemical groups, listed in Table 3. It was decided to treat all VOCs belonging to the different chemical groups jointly to provide analogous conditions as in the case of grain aroma, which may be recorded olfactometrically. On this basis, the significantly greatest abundance of VOCs was recorded in samples of grain collected from the fungicide-treated variant and the control (29% and 27%, respectively) amounting to 1.00 and 0.94 relative units, followed by the inoculated variant at 25% (0.86), while it was the lowest in the organic farming variant (18%, 0.63). The organic farming variant was also characterized by the significantly lowest total abundances for such groups as alcohols, benzenes, furan derivatives, ketones, and terpenes. On the other hand, phenylpropanoids constituted the only chemical group, for which the highest abundance was stated in the organic variant. Both inoculated variants (inoculated and fungicide) differed significantly not only in terms of the total VOCs content but also in individual chemical groups. Significant differences were found for such groups as aromatic hydrocarbons, esters, aliphatic hydrocarbons, nitrogen compounds, and sulfur compounds. It should also be noted that among those groups, only nitrogen compounds were found in significantly greater amounts in the inoculated variant. Another observation was related to the low variation of terpenes, between variants of the experiments (Table 3). Terpenes are a group of compounds of particular importance in cereals. Terpene analysis allows to indicate the presence of toxin-producing fungal strains. In order to identify VOCs differentiating the studied population, a discriminant analysis was performed, the results of which are presented in Figure 2. This approach uses stepwise linear discriminant analysis, where a classification model is built step by step. In each step, individual features are added (forward analysis) or eliminated (backward analysis) and their contribution to the classification is scored. The features that contribute best are then included into the discrimination function and the analysis proceeds with the next step.

Complete discrimination was obtained based on a model including 116 out of 146 VOCs. This analysis showed variation among the tested objects. Next, in order to provide a better separation, it was decided to include in the further analyses only 62 compounds with the greatest discriminatory power, which are given in Table 4. The results showed that 41 VOCs were found in the lowest amounts in the case of the organic variant, while the greatest numbers of compounds were recorded in the grain samples from the control (16 VOCs) and the inoculated variant (13 VOCs). It was also observed that among 62 compounds, an important role is played by VOCs indicated in literature sources as metabolites of microscopic fungi, such as, e.g., 1-octanol, 1-octen-3-ol, 2-heptanone, 2-nonanone, and trichodiene [22]. It needs to be stressed that these compounds, also considered to be precursors of mycotoxin biosynthesis, in samples of organic grain were found at the lowest abundance. In the case of the inoculated variant, they were the most abundant, which indicates their direct relationship with the development of pathogens. 

**Figure 3:**
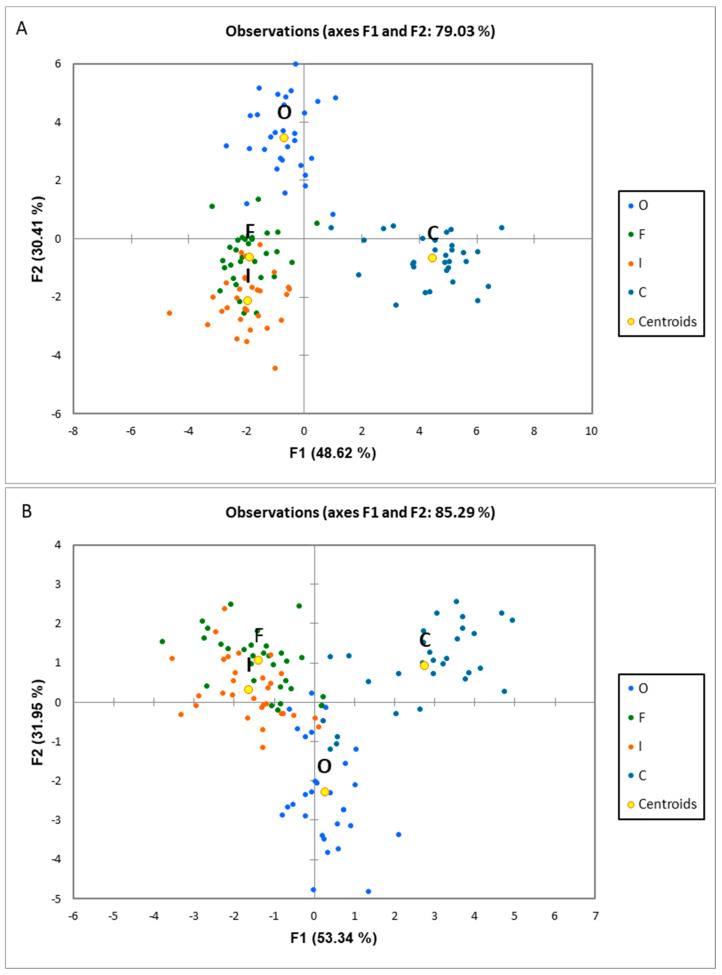
Results of discrimination analysis based on 37 (**A**) and 14 (**B**) compounds (listed below). Centroids of particular groups—average of observations of 30 analyzed varieties according to Table 1, O—organic, F—fungicide, I—inoculated, C—control. (**A**)—1-Butanol; 1-Hexanol; 1-Octanol; 1-Pentanol; 1-Penten-3-ol; 3-Octanol, 3,7-dimethyl-; Benzeneacetaldehyde; Hexanal, 2-ethyl-; Benzaldehyde, 4-ethyl-; Benzene, 1,3-dimethyl-5-(1-methylethyl)-; Benzene, 1,4-diethyl-; Benzophenone; Biphenylene; Phenol, 2,4-bis(1,1-dimethylethyl)-; 1,3,5,7-Cyclooctatetraene; Benzoic acid, 2-hydroxy-, pentyl ester; 2(3H)-Furanone, 5-ethenyldihydro-5-methyl-; 2(3H)-Furanone, 5-ethyldihydro-; 2(3H)-Furanone, 5-hexyldihydro-; Benzothiazole; Hexane; Octane, 3,5-dimethyl-; Tetradecane; 1-Penten-3-one; 2,6,6-Trimethyl-2-cyclohexene-1,4-dione; 2-Heptanone, 6-methyl-; 2-Hexanone; 2-Hexanone, 4-methyl-; 2-Nonanone; 2-Pentanone; 3-Ethylcyclopentanone; Cyclohexanone, 2,2,6-trimethyl-; Cyclohexanone, 5-methyl-2-(1-methylethyl)-; 1,3-Benzodioxole, 5-(2-propenyl)-; 2-Undecanethiol, 2-methyl-; 3-Cyclohexene-1-methanol, Ó,Ó4-trimethyl-; Trichodiene. (**B**)—1-Butanol; 1-Methylcycloheptanol; 3-Pentanol, 2-methyl-; Benzaldehyde, 4-ethyl-; Benzene, 1,3-dimethyl-5-(1-methylethyl)-; 1,3,5,7-Cyclooctatetraene; 2(3H)-Furanone, 5-ethenyldihydro-5-methyl-; Hexane; 2-Hexanone, 4-methyl-; 2-Nonanone; 3-Buten-2-one, 4-phenyl-, (E)-; 1,3-Benzodioxole, 5-(2-propenyl)-; 2-Undecanethiol, 2-methyl-; Benzenemethanol, Ó,Ó,4-trimethyl-.

In the next step of discriminant analysis, the lowest numbers of VOCs differentiating the investigated experimental populations were determined by applying the backward and forward analyses, which identified 37 and 14 VOCs, respectively, thus facilitating discrimination of the tested variants (Figure 3A,B).

**Figure 4:**
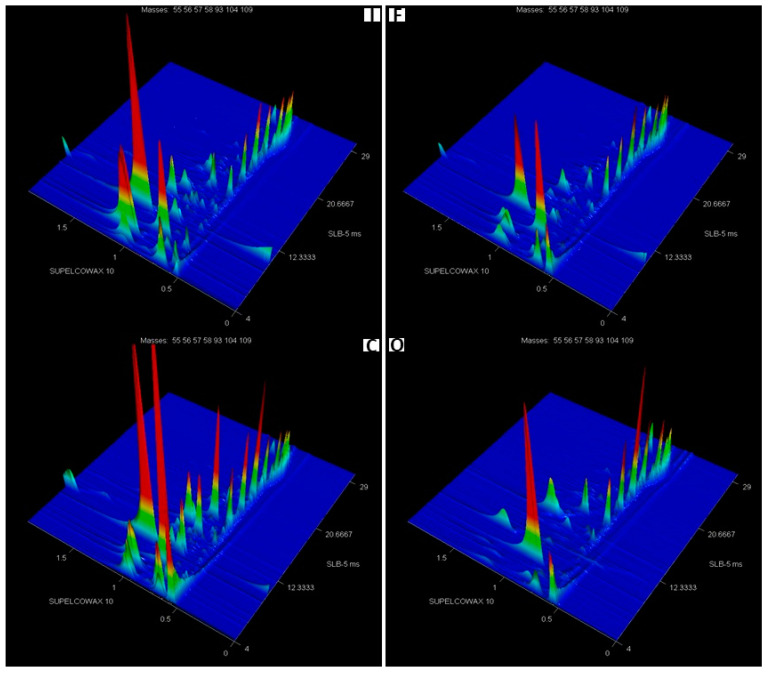
GC × GC-ToFMS chromatogram of volatile compounds typical of fungi, obtained from wheat grain (cultivar Kampana) from inoculated (I), fungicide (F), control (C), and organic (O) variant of the experiment. The same peak intensity scale is used for all chromatograms.

Analysis of the results indicates that in the cases of discriminant analysis based on 37 and 14 compounds, samples from the organic variant differ markedly from the samples in the other variants, while the fungicide treatment and the inoculated variants are located close. However, the inoculated variant in the model including 116 VOCs was also significantly separated. In the case of the model comprising 37 compounds, 4 of them may be classified as fungal compounds, whereas in the model consisting of 14 VOCs, 2 of them may be classified as fungal compounds (Table 5). Given the above and because inoculation was applied in two experimental variants, it was decided to separately analyze these metabolites, which are related particularly to the development of fungal pathogens, as presented in example chromatograms of ions characteristic of these fungal compounds (Figure 4). Analyses of the presented chromatograms showed varied abundance for individual experimental variants. The highest peaks were observed after inoculation, followed by the variant using fungicides, similar to the control. Markedly smaller peaks were recorded in the case of organic farming.

Among all the identified compounds being metabolites of microscopic fungi, five were detected in the smallest amounts in grain samples from the organic variant. Among these compounds, a particularly important role is played by trichodiene. This trichothecene precursor was found at the highest concentration in grain samples from the inoculated variant. In contrast, no significant differences were observed in the abundance of trichodiene between samples from the fungicide-treated variant and the control. Next to trichodiene in the samples of the inoculated variant, the significantly highest abundance was recorded for 2-heptanone and 2-nonanone, i.e., ketone compounds.

A problem that needs to be fully investigated is connected with the potential link between the VOCs found in grain and resistance to fungal diseases in cultivars included in the experiment. For this purpose, discriminant analysis was conducted to characterize the tested winter wheat cultivars in terms of their resistance to FHB (Table 1). The analysis was conducted based on 116 VOCs and the cultivars were divided into 4 groups: R—resistant; MR—medium resistant; MS—medium susceptible; and S—susceptible to ear blight. The presented results indicate a complete separation of all four groups. We need to focus particularly on the very large distance between groups R and S. In contrast, the groups characterized by medium resistance and susceptibility were distributed within a small distance from each other.

## 4. Discussion

The presented experiment was conducted in four experimental variants, of which two were artificially inoculated with *Fusarium culmorum*, while two were naturally colonized by microorganisms. The organic farming variant was unique, as no chemical pesticides were applied here.

The applied advanced method of VOCs analysis comprised two-dimensional chromatographic separation, as described in detail in the Materials and Methods. Such an approach provides a markedly better separation of compounds than the routinely used traditional single-column separation [23]. In combination with the time-of-flight mass spectrometry, it facilitated the identification of 146 VOCs, which, taking into consideration the analysis of samples of grain growing under field conditions, constitutes significant progress in comparison to earlier analyses [22]. However, because of the chemical character of identified compounds, it may be stated that they belonged to the same chemical groups as those detected previously [24]. Studies on VOCs both in cereals and fungi, of which a considerable number constitute the natural phyllosphere of grain but also comprise pathogens, have been conducted for many years both by other researchers and our team [24,25]. The identified VOCs include compounds from such chemical groups as alcohols, aldehydes, ketones, benzenes, cyclic compounds, esters, furan derivatives, heterocyclic compounds, hydrocarbons, nitrogen compounds, phenolic compounds, phenylpropanoids, sulfur compounds, and terpenes. Based on literature data describing fungal VOCs, particularly those formed by *Fusarium*, the relatively poor profile of terpenes in the grain samples analyzed within this study is an interesting finding. We need to stress here that in most scientific papers on the subject, the presented terpene profile was derived from cultures [26,27,28]. As stated above, the profile of VOCs produced by fungi is significantly affected by the conditions in which they grow [26]. In our earlier studies, it was also shown that individual VOCs belonging to the terpene group show variation depending on whether the development of fungi from the genus *Fusarium* was mass-scale (infection or inoculation). In this respect, the chemotype of *Fusarium* may be of importance, while to date, it has been most frequently investigated under laboratory conditions [26]. In contrast, data obtained in this study came from the analysis of samples of grain grown under field conditions.

It is worth noting that only four VOCs (1-methylcycloheptanol, 2-heptanone, 2(3H)-furanone, 5-hexyldihydro-2(3H)-furanone) appeared in statistically significant differences in all four experimental variants. In the case of 1-methylcycloheptanol, it is difficult to find literature data describing its importance in plants. In turn, 2-heptanone is created by bacteria that inhabit beehives. This compound has a protective role against honeybee pathogens [29]. Furanones are compounds produced by plants and have antibacterial properties [30].

It seems that among the obtained data, the most important are those describing the profile of VOCs in the grain of wheat grown in the organic system, since available literature on the subject lacks results concerning VOCs from grain produced in that cultivation system. The analysis of the VOCs profiles in grain of wheat from four experimental variants showed marked differences between samples of grain grown in the organic system and the other variants. An interesting observation is connected with the unique VOCs profile of grain from the organic variant, differing from the inoculated variants (inoculated and fungicide treated), and also considerable quantitative differences in relation to the control variant. In all these cases, grain coming from the organic system was characterized by the lowest abundance of VOCs. This observation is surprising, particularly since the surface of grain from wheat grown in the organic system is typically colonized by microbiota to a greater extent than grain from conventional cultures [31]. In this experiment, this fact was already preliminarily confirmed during earlier studies [32]. However, the key finding is connected with a significant share of probiotic microorganisms, whose development is limited through competition by the potential mass-scale development of pathogenic microbiota. However, it was also stated that grain produced in the organic cultivation system is smaller than that grown in the traditional system and it contains less fat. Analyses of the biochemical pathways of VOCs formation show a considerable role in changes related to fatty acid metabolism [33]. The lower availability of fat substrates at the simultaneous greater total population of microbiota colonizing kernel surface may have resulted in lower VOCs levels in grain from the organic variant. In terms of the development of fungal pathogens, we also need to stress particularly the lowest abundance of trichodiene in grain of the organic variant. Trichodiene as a precursor of trichothecenes has also been proposed as a marker of the development of toxigenic *Fusarium* fungi [12,27]. As was shown by Perkowski et al. [34], this marker is present also in non-inoculated grain, where fungi from the genus *Fusarium* may develop as a result of natural infection. However, the conditions for the mass-scale attack of toxigenic *Fusarium* strains are characterized by a marked dominance of trichodiene in the terpene profile of infested grain and occasionally also the total VOCs profile [22]. The lowest mean trichodiene content was recorded in the case of grain samples from the O variant discussed in this study. This clearly shows limited growth potential for pathogenic fungi in grain grown in the organic system. It results not only from the reduced living space due to the presence of probiotic microorganisms and enhanced plant immune responses [32], but also the presence of certain VOCs. The latter include branched chain alcohols such as butanol, 3-methyl or butanol, 2-methyl [35]. The first of the above-mentioned compounds was detected in grain samples analyzed in this study. The greatest abundance of this metabolite was characteristic of grain in the fungicide-treated variant, while grain from the organic variant contained its lowest amounts. An in-depth analysis of biochemical changes connected with branched-chain alcohols reveals their considerable complexity [36]. These compounds are formed directly from respective aldehydes, which in turn are derived from the metabolism of amino acids—leucine, but also threonine. Leucine may be formed as a result of protein distribution, but its biosynthesis may be related to fatty acids through pyruvate. Branched-chain alcohols may be further oxidized to carboxylic acids and esterified. The greatest abundance of butanol, 3-methyl in grain from the fungicide-treated variant may be caused directly by the application of fungicides during cultivation. Many of them interfere with the biosynthesis of fungal sterols [37]. Still, fungicide treatment is a factor disturbing the equilibrium in kernel microbiota. This disturbance will be reflected in the VOC profile, as evidenced by the highest total abundance of VOCs in grain of this variant.

Another factor, which also affects the development of microbiota on kernel surface and the production of their metabolites, is connected with the application of nitrogen fertilization [38]. In the case of most mycotoxins, it was shown that a greater dose of nitrogen in wheat fertilization caused a significant increase in the accumulation of mycotoxins compared to cultures, in which lower nitrogen doses were applied. Moreover, the cultivar x nitrogen dose interaction was found to have a significant effect leading to markedly higher mycotoxin contents in wheat [39].

A significant element of this study, not reported in available literature on the subject, was connected with the analysis of VOCs in grain taking into consideration the characteristics of tested winter wheat cultivars in terms of their resistance to FHB. The conducted discriminant analysis (data not presented) showed a surprising finding, i.e., the potential complete separation of individual groups of wheat in terms of their resistance to FHB based on VOCs. Thus, this result confirms the applicability of VOCs analysis to evaluate winter wheat grain for this trait. It may also prove useful for cereal breeders in identifying cultivars taking into consideration their resistance traits.

## 5. Conclusions

In conclusion, this study showed a significantly different profile of VOCs in grain from organic farming in relation to VOCs for all the tested experimental variants. In the case of three VOCs (1-methylcycloheptanol, 2-heptanone, 2(3H)-furanone, 5-hexyldihydro-2(3H)-furanone), a statistically significant difference was observed between all the four experimental variants. Moreover, a direct relationship was found between VOCs characteristic of fungal metabolism and the development of fungal pathogens. An example is the presence of the smallest amount of trichodiene in the organic variant. It is also important to demonstrate, on the basis of discriminant analysis, that the organic variant is significantly different from other variants. Thus, it provides the foundations for further in-depth studies on this problem. Both these results and the team’s previous results regarding the use of VOCs analysis to assess the quality of cereal grain due to infection by toxigenic fungal pathogens allow attempts to create a quality assessment system in field and storage conditions.

## Figures and Tables

**Table 1:** List of winter wheat cultivars used in this study. Classes of Fusarium head blight resistance: susceptible (S), medium susceptible (MS), medium resistant (MR), and resistant (R).

No.	Cultivar		No.	Cultivar		No.	Cultivar	
1	Akteur	MS	11	Jenga	MS	21	Naridana	MS
2	Alcazar	S	12	Kampana	S	22	Nateja	R
3	Anthus	MS	13	Kohelia	MR	23	Ostka Strzelecka MS	
4	Batuta	MS	14	Legenda	MR	24	Ostroga	MR
5	Belenus	MS	15	Ludwig	MS	25	Slade	MS
6	Bogatka	MR	16	Markiza	MS	26	Smuga	S
7	Boomer	MR	17	Meteor	MS	27	Sukces	MR
8	Dorota	MR	18	Mewa	MS	28	Tonacja	MR
9	Figura	MS	19	Mulan	MS	29	Türkis	MS
10	Garantus	MS	20	Muszelka	S	30	Zyta	MR

**Table 2:** Temperature (average, minimal, maximal) and precipitation in Radzików for May, June, and July 2020.

Months	May		June		July	
Temperature °C						
	Min	Max	Min	Max	Min	Max
I decade	5.2	17.9	10.7	21.6	14.1	26.4
II decade	4.6	16.8	15.4	26.4	12.2	24.8
III decade	6.8	18.8	16.6	26.3	12.9	26.2
Average	5.6	17.8	14.2	24.7	13.1	25.8
Average temperature	11.8		19.1		19.2	
Precipitation (mm)						
I decade	18.8		16.0		0.6	
II decade	24.4		34.4		4.0	
III decade	30.8		2.9		6.6	
Sum of precipitation	74.0		53.3		11.2	

**Table 3:** Results of the volatile compounds (in peak area abundance × 10^7^) of VOCs of particular chemical groups of the three repetitions of 30 cultivars of winter wheat grain in four variants of the experiment; O—organic, F—fungicide, I—inoculated, C—control.

Chemical Group	O		F		I		C	
Alcohol	221.61	^b^	515.01	^a^	427.88	^a^	471.33	^a^
Aldehyde	156.11	^b^	245.69	^a^	202.15	^ab^	182.96	^ab^
Aromatic hydrocarbons	32.90	^c^	49.28	^a^	41.99	^b^	45.54	^ab^
Cyclic compounds	259.31	^b^	241.66	^b^	276.15	^b^	349.16	^a^
Esters	94.09	^ab^	128.43	^a^	88.34	^b^	91.11	^ab^
Furan derivatives	64.23	^c^	120.39	^ab^	140.43	^a^	101.20	^b^
Heterocyclic compounds	108.56	^ab^	112.93	^a^	161.01	^a^	120.47	^a^
Aliphatic hydrocarbon	116.41	^b^	228.87	^a^	99.53	^b^	232.55	^a^
Ketone	70.70	^b^	108.94	^a^	110.91	^a^	103.62	^a^
Nitrogen compounds	7.49	^bc^	6.92	^c^	9.43	^a^	8.22	^b^
Phenolic compound	4.58	^a^	4.44	^a^	4.09	^a^	5.05	^a^
Phenylpropanoids	0.63	^a^	0.24	^c^	0.20	^c^	0.38	^b^
Sulfur compound	4.10	^b^	58.89	^a^	4.21	^b^	2.92	^b^
Terpene	28.43	^b^	37.59	^a^	36.85	^a^	35.25	^a^
Σ	1169.15	^c^	1859.29	^a^	1603.17	^b^	1749.75	^a^

The same letters in the same row indicate a lack of significant differences between values according to one-way ANOVA (significant level 95%, variants of the experiment as the factor and varieties as the repetition). The occurrence of two or more letters (“^ab^”, “^bc^”) indicate a lack of significant differences between values described as “^a^” and “^b^” or “^a^”, “^b^” and “^c^” according to one-way ANOVA.

**Table 4:** Results of the discriminant analysis based on 62 VOCs with the highest discriminant power (in peak area abundance (×10^7^) of the three repetitions of 30 cultivars of winter wheat grain in four variants of the experiment; O—organic, F—fungicide, I—inoculated, C—control.

Chemical Group	Compound	O		F		I		C	
Alcohol	1-Butanol *†	34.383	^c^	51.170	^a^	36.418	^c^	43.881	^b^
	1-Heptanol, 2-propyl-	1.546	^c^	2.696	^b^	2.143	^bc^	4.831	^a^
	1-Hexanol *	76.887	^b^	189.240	^a^	165.576	^a^	191.308	^a^
	1-Methylcycloheptanol †	0.399	^d^	1.461	^b^	2.106	^a^	0.769	^c^
	1-Octanol *	0.591	^b^	3.154	^a^	2.944	^a^	2.587	^a^
	1-Octen-3-ol	6.649	^c^	17.572	^a^	17.936	^a^	12.644	^b^
	1-Pentanol *	34.617	^c^	75.650	^a^	62.998	^b^	55.990	^b^
	1-Penten-3-ol *	12.972	^c^	22.353	^b^	18.259	^bc^	33.323	^a^
	3-Octanol, 3,7-dimethyl- *	0.586	^c^	0.977	^b^	1.223	^a^	1.027	^b^
	3-Pentanol, 2-methyl- †	1.810	^bc^	2.935	^a^	1.503	^c^	2.259	^b^
	Benzenemethanol, α,α-dimethyl-	0.423	^c^	0.616	^b^	0.590	^b^	0.787	^a^
	Ethanol, 2-butoxy-	4.614	^d^	8.002	^b^	6.230	^c^	9.270	^a^
Aldehyde	Benzeneacetaldehyde *	5.084	^c^	8.790	^b^	11.335	^a^	9.611	^b^
	Butanal, 2-ethyl-3-methyl-	1.553	^c^	2.852	^a^	2.416	^b^	2.373	^b^
	Hexanal, 2-ethyl- *	0.986	^c^	1.718	^a^	1.614	^a^	1.376	^b^
Aromatic hydrocarbons	1-(3-Methylbutyl)-2,3,4-trimethylbenzene	0.243	^b^	0.336	^a^	0.370	^a^	0.323	^a^
	1H-Indene, 1-ethylidene-	6.253	^b^	11.032	^a^	10.498	^a^	11.021	^a^
	Benzaldehyde, 4-ethyl- *†	2.431	^a^	0.572	^bc^	0.344	^c^	0.979	^b^
	Benzene, 1,2,3-trimethyl-	3.824	^c^	5.148	^b^	5.638	^ab^	5.767	^a^
	Benzene, 1,3-dimethyl-5-(1-methylethyl)- *†	0.226	^c^	0.327	^b^	0.241	^c^	0.736	^a^
	Benzene, 1,4-diethyl- *	0.418	^c^	0.643	^b^	0.738	^a^	0.698	^ab^
	Biphenyl	2.171	^b^	2.967	^a^	3.275	^a^	2.993	^a^
	Biphenylene *	0.440	^b^	0.719	^a^	0.665	^a^	0.694	^a^
	Naphthalene, 1,2,3,4-tetrahydro-	0.208	^b^	0.283	^a^	0.298	^a^	0.294	^a^
	Naphthalene, 1,2,3-trimethyl-4-propenyl-, (E)-	0.153	^c^	0.250	^a^	0.158	^c^	0.203	^b^
	Phenol, 2,4-bis(1,1-dimethylethyl)- *	0.207	^c^	0.443	^a^	0.327	^b^	0.448	^a^
Cyclic compounds	1,3,5,7-Cyclooctatetraene *†	14.861	^b^	17.715	^b^	20.745	^b^	69.634	^a^
Esters	Benzoic acid, 2-hydroxy-, pentyl ester *	0.171	^c^	0.213	^b^	0.225	^b^	0.266	^a^
	Benzoic acid, 4-(1-methylethyl)-, methyl ester	0.872	^c^	1.626	^ab^	1.782	^a^	1.312	^b^
Furan derivatives	2(3H)-Furanone, 5-ethenyldihydro-5-methyl- *†	0.753	^b^	0.872	^b^	0.856	^b^	1.100	^a^
	2(3H)-Furanone, 5-ethyldihydro- *	6.083	^b^	9.844	^a^	10.542	^a^	9.883	^a^
	2(3H)-Furanone, 5-hexyldihydro- *	2.793	^d^	5.959	^b^	7.904	^a^	5.031	^c^
	Dibenzofuran	2.247	^b^	3.400	^a^	3.172	^a^	3.517	^a^
Heterocyclic compounds	Benzothiazole *	2.125	^c^	3.737	^b^	3.751	^b^	4.367	^a^
	Oxepine, 2,7-dimethyl-	0.654	^c^	0.865	^b^	0.937	^b^	1.092	^a^
Aliphatic hydrocarbon	Decane, 2-methyl-	0.875	^c^	1.227	^b^	0.959	^bc^	2.615	^a^
	Hexane *†	62.511	^b^	154.362	^a^	17.947	^c^	157.257	^a^
	Octane, 3,5-dimethyl- *	1.636	^d^	2.653	^b^	3.133	^a^	2.258	^c^
	Tetradecane *	20.006	^c^	21.870	^c^	25.761	^b^	30.290	^a^
	Undecane, 5-ethyl-	3.068	^c^	4.163	^b^	4.834	^a^	3.568	^c^
Ketone	1-Penten-3-one *	5.829	^a^	4.148	^b^	2.231	^c^	4.354	^b^
	2,6,6-Trimethyl-2-cyclohexene-1,4-dione *	1.178	^c^	1.917	^b^	1.770	^b^	2.341	^a^
	2-Cyclohexen-1-one, 3,5,5-trimethyl-	0.589	^c^	0.880	^b^	1.054	^a^	1.002	^ab^
	2-Heptanone	8.130	^d^	16.893	^b^	20.269	^a^	13.672	^c^
	2-Heptanone, 6-methyl- *	3.155	^c^	4.603	^b^	5.414	^a^	4.476	^b^
	2-Hexanone *	1.799	^b^	2.916	^a^	3.337	^a^	3.038	^a^
	2-Hexanone, 4-methyl- *†	1.447	^b^	0.748	^c^	0.918	^c^	1.997	^a^
	2-Nonanone *†	2.850	^c^	4.049	^b^	5.977	^a^	3.609	^bc^
	2-Pentanone *	9.061	^b^	17.851	^a^	17.222	^a^	15.693	^a^
	3-Buten-2-one, 4-phenyl-, (E)- †	0.998	^b^	0.613	^c^	1.351	^a^	1.157	^ab^
	3-Ethylcyclopentanone *	0.581	^b^	0.862	^a^	0.817	^a^	0.928	^a^
	3-Heptanone	2.564	^c^	4.215	^b^	4.218	^b^	5.619	^a^
	Benzophenone *	0.322	^b^	0.534	^a^	0.384	^b^	0.550	^a^
	Cyclohexanone, 2,2,6-trimethyl- *	0.366	^c^	0.611	^b^	1.000	^a^	0.675	^b^
	Cyclohexanone, 5-methyl-2-(1-methylethyl)- *	0.202	^c^	0.294	^bc^	0.535	^a^	0.408	^b^
Phenylpropanoids	1,3-Benzodioxole, 5-(2-propenyl)- *†	0.631	^a^	0.241	^c^	0.204	^c^	0.377	^b^
Sulfur compound	2-Undecanethiol, 2-methyl- *†	1.019	^c^	4.336	^a^	2.329	^b^	1.553	^bc^
Terpene	2,5-Cyclohexadiene-1,4-dione, 2,6-bis(1,1-dimethylethyl)-	0.172	^c^	0.228	^b^	0.283	^a^	0.240	^b^
	3-Cyclohexene-1-methanol, Ó,Ó4-trimethyl- *	0.539	^b^	0.998	^a^	1.052	^a^	0.983	^a^
	Benzenemethanol, Ó,Ó,4-trimethyl- †	1.379	^c^	2.793	^b^	2.768	^b^	3.542	^a^
	Bicyclo[3,1,1]hept-2-ene, 2,6,6-trimethyl-, (˝)-	6.866	^c^	10.668	^a^	10.696	^a^	8.495	^b^
	Trichodiene *	0.985	^c^	1.687	^b^	2.662	^a^	1.521	^b^

* VOCs (37) included in the model based on Backward DA, † VOCs (14) included in the model based on Forward DA. The same letters in the same row indicate a lack of significant differences between values according to one-way ANOVA (significance level 95%, variants of the experiment as the factor and varieties as the repetition).

**Table 5:** Fungal volatile organic compounds in peak area abundance (×10^7^) of the tree repetitions of 30 cultivars of winter wheat grain in four variants of the experiment; O—organic, F—fungicide, I—inoculated, C—control.

Compound	O		F		I		C	
1-Butanol, 3-methyl-	17.24	^c^	90.76	^a^	59.57	^b^	59.75	^b^
1-Octanol	0.59	^b^	3.15	^a^	2.94	^a^	2.59	^a^
1-Octen-3-ol	6.65	^c^	17.57	^a^	17.94	^a^	12.64	^b^
Hexanal	55.40	^b^	104.65	^a^	65.84	^ab^	51.00	^b^
Octanal	5.32	^a^	9.11	^a^	5.18	^a^	9.20	^a^
1,3,5,7-Cyclooctatetraene	14.86	^b^	17.72	^b^	20.75	^b^	69.63	^a^
2-Heptanone	8.13	^d^	16.89	^b^	20.27	^a^	13.67	^c^
2-Nonanone	2.85	^c^	4.05	^b^	5.98	^a^	3.61	^bc^
2-Octanone	6.57	^c^	20.33	^a^	14.85	^ab^	10.90	^bc^
3-Carene	1.40	^a^	1.22	^a^	0.74	^b^	1.55	^a^
Trichodiene	0.98	^c^	1.69	^b^	2.66	^a^	1.52	^b^

The same letters in the same row indicate a lack of significant differences between values according to one-way ANOVA (significance level 95%, variants of the experiment as the factor and varieties as the repetition).

## Data Availability

Data is contained within the article.
